# The Role of Computer-Assisted Technology in Post-Traumatic Orbital Reconstruction: A PRISMA-driven Systematic Review

**DOI:** 10.1038/srep17914

**Published:** 2015-12-08

**Authors:** Kelvin H. Wan, Kelvin K. L. Chong, Alvin L. Young

**Affiliations:** 1 Department of Ophthalmology, Tuen Mun Eye Center and Tuen Mun Hospital, Hong Kong; 2 Department of Ophthalmology and Visual Sciences, Prince of Wales Hospital; 3 Alice Ho Liu Ling Nethersole Hospital, Hong Kong; 4 Department of Ophthalmology and Visual Sciences, the Chinese University of Hong Kong, Hong Kong.

## Abstract

Post-traumatic orbital reconstruction remains a surgical challenge and requires careful preoperative planning, sound anatomical knowledge and good intraoperative judgment. Computer-assisted technology has the potential to reduce error and subjectivity in the management of these complex injuries. A systematic review of the literature was conducted to explore the emerging role of computer-assisted technologies in post-traumatic orbital reconstruction, in terms of functional and safety outcomes. We searched for articles comparing computer-assisted procedures with conventional surgery and studied outcomes on diplopia, enophthalmos, or procedure-related complications. Six observational studies with 273 orbits at a mean follow-up of 13 months were included. Three out of 4 studies reported significantly fewer patients with residual diplopia in the computer-assisted group, while only 1 of the 5 studies reported better improvement in enophthalmos in the assisted group. Types and incidence of complications were comparable. Study heterogeneities limiting statistical comparison by meta-analysis will be discussed. This review highlights the scarcity of data on computer-assisted technology in orbital reconstruction. The result suggests that computer-assisted technology may offer potential advantage in treating diplopia while its role remains to be confirmed in enophthalmos. Additional well-designed and powered randomized controlled trials are much needed.

Orbital fractures occur in 40% of all craniomaxillofacial traumas^1^,^2^. Patients often present with diplopia or enophthalmos for surgical repair^3^. Enophthalmos, clinically defined as an inter-ocular difference of more than 2 mm in exophthalmometry, remains a surgical challenge in post-traumatic orbital reconstruction^4^,^5^,^6^,^7^. Orbital fracture repair includes reducing prolapsed tissues, removing unstable bones, and replacing the bony defect with an implant without hindering extraocular motility^8^.

Computer-assisted technology using computerized tomography (CT) plays an emerging role in orbital reconstruction during preoperative planning, implant design, intraoperative navigation and postoperative auditing. In preoperative planning, a mirror image overlay (MIO) is created based on the uninjured, contralateral orbit and is superimposed onto the images of the injured side^9^. During computer-aided design and computer-aided modeling (CAD/CAM), the CT data is segmented and digitally transformed to create a three-dimensional model via stereolithography^10^,^11^. This is then used to manually mould or manufacture a “patient-specific” implant^12^,^13^. Using intra-operative navigation, surgeons obtain real-time coordinates of surgical instruments and implants with respect to the surrounding bony structures.

While there are recommendations and reviews on the indication and timing of surgery^3^, materials of reconstruction^14^, the role of endoscopic-assisted repair^15^, the role of computer-assisted technology in orbital reconstruction has not been defined. To address this, we conducted a systematic review on the use of computer-assisted technology augmented surgery versus conventional surgery in post-traumatic orbital reconstruction to evaluate its functional and safety outcomes.

## Methods

### Eligibility criteria for considering studies for this review

Studies were included if they:

1. Compared computer-assisted technology versus conventional surgery.
2. Used computer-assisted technology in the treatment of orbital reconstruction, including but not limited to preoperative planning, surgical navigation, or CAD/CAM implants.
3. Included detailed description of the computer-assisted techniques used.
4. Performed primary and/or secondary reconstruction of fractured orbital walls.
5. Examined at least one of the following outcomes: diplopia, enophthalmos, or procedure-related complications.

Studies were excluded if:

1. Computer-assisted technology were only used for diagnostic purposes.
2. Orbital reconstruction for non-traumatic (e.g. post tumor removal) cases.
3. Outcomes were reported in a qualitative manner.
4. Preoperative or postoperative outcome measurements were inadequate.

### Search methods for identifying studies

We searched MEDLINE and EMBASE via the OVID platform, Cochrane Central Register of Controlled Trials (CENTRAL), ClinicalTrials.gov (www.clinicaltrials.gov) and World Health Organization International Clinical Trials Registry Platform (WHO ICTRP) for any comparative studies. We used the Cochrane highly sensitive search strategy and combined with our search terms^16^. The detailed search strategy can be found in the Appendix 1 (supplementary material). We searched the references of the retrieved full-text articles to identify studies not found by our search strategy. We did not apply any language restriction. The final search was performed on February 17, 2015 for all the databases.

### Study selection and Data Collection

Two reviewers (KHW and KKLC) independently assessed the titles and abstracts. In case of any unresolved discrepancies, a third reviewer (ALY) will arbitrate until a mutual conclusion was reached. We used a customized form to record the authors, year of publication, sample size, duration of follow up, fracture patterns, primary or secondary reconstruction, the modality of computer-assisted technologies used, surgical techniques, implants, and outcome measures. We collected outcome data at the last follow-up. We used reduction in enophthalmos measured by exophthalmometer in millimeter (mm); if these data were not available, then the percentage of patients showing enophthalmos at the last follow-up were analyzed. Likewise, we extracted the percentage of patient complaining of diplopia at the last follow-up. Number and types of complications were recorded.

### Risk of Bias Assessment

The quality of the studies was independently evaluated by 2 reviewers (KHW and KKLC) using the Cochrane Risk of Bias tools for RCTs^17^ and the Newcastle-Ottawa Scale for the observational studies^18^.

### Statistical Analysis

We used the Review Manager (RevMan, version 5.3; Copenhagen: The Nordic Cochrane Centre, The Cochrane Collaboration, 2014) for our meta-analysis. We analyzed the statistical heterogeneity of the included studies with the Cochrane Q-statistics chi-square test and I^2^ statistic. If there was any significant heterogeneity between studies (p < 0.1), a random-effect model was used for pooling the data; otherwise a fixed-effect model was used.

## Results

**Figure 1:**
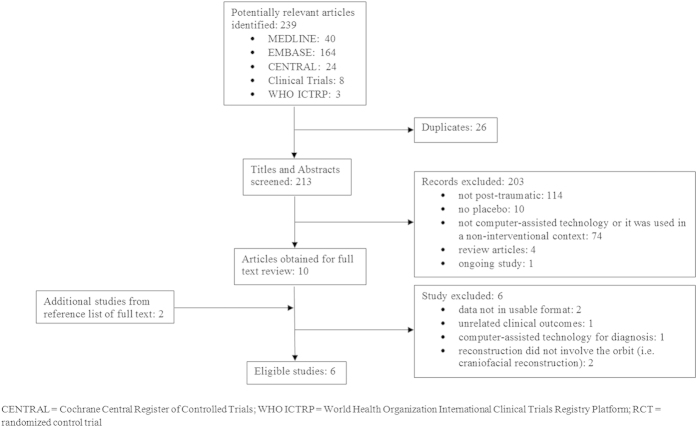
Flow chart showing selection of publications for inclusion in this systematic review.

We identified 239 titles and abstracts through our literature search and retrieved 12 articles for full text review. We included 6 studies in this systematic review according to our a priori criteria (Fig. 1)^19^,^20^,^21^,^22^,^23^,^24^. The PRISMA checklist and flow diagram can be found in Appendices 2 and 3 (supplementary material).

### Study Characteristics

Table 1 shows the characteristics of the included trials. We did not identify any randomized controlled trials. Of the identified articles, 4 were prospective cohort studies^20^,^21^,^22^,^23^. 1 was a historical cohort trial^19^ and 1 was a retrospective cohort study^24^. Two studies described isolated orbital floor fracture^22^,^24^ while the rest had patients involving multiple or different orbital walls. Patients with secondary orbital reconstructions were examined in 2 studies^21^,^22^.

Table 2 summarizes the details of the 6 included studies. Computer-assisted intra-operative surgical navigation was studied in three studies using non-preformed implants of different materials^19^,^20^,^21^; two of which also applied the MIO technique during surgical planning^19^,^20^. Preformed CAD/CAM implants using glass-bioceramic or titanium mesh were evaluated in the other 3 studies^22^,^23^,^24^, which none of these studies used intra-operative navigation device.

### Quality of Included Studies

Tables 3 and 4 summarize the risk of bias assessment of the included studies. Only 1 study used masked outcome assessor and addressed the confounding factors by matching the study and control group for age, sex, fracture pattern, preoperative ophthalmic features, etiology and severity of trauma, surgical approach and types of implant used^20^. The control groups were not comparable in two studies leading to potential bias on treatment effect^22^,^24^. Intervention bias was minimized in 2 studies by involving only one surgeon^19^,^22^. All studies had appropriate length of follow up and accounted for missing data.

### Outcome Measures

Tables 5 and 6 summarize the outcomes of the three studies on intraoperative surgical navigation and the other three on individualized preformed CAD/CAM implants group, respectively. Four studies reported on postoperative diplopia^19^,^20^,^21^,^24^. Three of the 4 studies demonstrated statistically significant improvement of diplopia in the computer-assisted group over the control group (51 vs 60%^19^, 2 vs 10%^20^, 17 vs 88%^24^). Five studies measured changes of enophthalmos^20^,^21^,^22^,^23^,^24^. In the computer-assisted group, 3–27% of patients were still enophthalmic at the last follow-up, compared with 10–50% in the control group. A range of 1.5–3.25 mm improvement of enophthalmos was reported in the assisted group, compared to 1.8–3.88 mm in the control group. Four of these five studies concluded there was no difference in improving enophthalmos between the study group and the control group^20^,^21^,^22^,^23^. Only 1 study reported significantly lower percentage of patients with persistent enophthalmos using preformed CAD/CAM titanium mesh implants as compared to calvarial bone grafts^24^.

Procedure-related complications were documented in 3 studies^19^,^20^,^21^,^22^. In one study, extrusion or infection requiring implant removal, entropion, ectropion and epiphora were observed in the group using computer-assisted intra-operative surgical navigation, whereas implant removal, eyelid abscess, retrobulbar hematoma were encountered in the control^19^. In another study comparing preformed CAD/CAM glass-bioceramic implants implant with non-preformed titanium meshes, 1 case of reduced vision and 2 cases of suspected retrobulbar hematoma versus 2 cases of suspected retrobulbar hematoma were identified, respectively^22^. One study did not encounter any post-operative complications in either group^21^. The overall complication rates by individual studies were comparable between both groups.

## Discussion

Computer assisted surgery (CAS) (ICD-9-CM Intervention code 00.3) represents surgical concepts and methods utilizing computer technology for preoperative planning and intraoperative guidance. It includes image-guided navigation (IGN) and image-guided surgery (IGS). Such guidance improves accuracy of surgical gestures and reduces surgeon’s action and effort^25^. This helps to decrease surgical errors and reduce operating time^26^. Computer-aided design and computer-aided modeling (CAD/CAM) and rapid prototyping (RP) technologies use image-derived data to fabricate custom-made medical guides and implants^4^. The accuracy of CAS is affected by the resolution of the imaging data set, the precision of the computer algorithm and the accuracy during data registration using superficial bony and surface landmarks^27^,^28^. Facial swelling after acute trauma may limit the accuracy of the registration process but can be overcome using a combination of referencing methods^29^. In patients with unilateral trauma, mirroring the uninjured orbit onto the affected side further improves accuracy^9^,^26^. However, MIO assumes perfect symmetry while variations do exist between normal orbits^30^.

Intra-operative navigation provides real-time feedback of anatomical location of the tracking device with respect to preoperative images^25^. Locations of and distances to extraocular muscles, infraorbital and optic nerves as well as orbital fissures can be assessed when dissecting through prolapsed orbital soft tissues to minimize iatrogenic injury^31^. Surgeons can also confirm the location of implant after placement^32^. Individually or industrially preformed CAD/CAM implants are designed to reproduce the intricate three-dimensional shape of the preinjured orbit for immediate loading. These implants reduce the time for intraoperative manipulation such as bending, trimming and repetitive fittings^4^ and minimize surgical trauma to periorbital soft tissues^4^,^7^.

### Quality of Evidence

We did not identify any randomized controlled trials in computer-assisted technology for post-traumatic orbital reconstruction. Results of our systematic review were limited to cohort and case-control studies. Our review highlights the scarcity of relevant literatures. Clinical heterogeneities were present for the severities, location and number of fractures, primary versus secondary reconstruction, timing of surgery and surgical approaches including use of intraoperative navigation, implant types and materials. Methodological heterogeneities due to different type of study designs and operative approaches and clinical heterogeneities due to divergent patient groups limited us from performing meta-analysis.

### Efficacy and Safety

Three studies reported the number of patients with clinically significant enophthalmos (difference >2 mm)^20^,^21^,^24^, while 2 studies provided exophthalmometry measurements^22^,^23^. We were unable to demonstrate any added benefit in correcting enophthalmos with the use of computer-assisted technologies. In the only study that showed such benefit^24^, the authors used pre-fabricated titanium implants in the assisted group versus calvarial bone grafts in control^24^. The main drawbacks of bone graft include difficulty in contouring and variability in resorption^27^, while titanium mesh are flexible yet rigid and stable over time^33^,^34^,^35^. The differences in implants used may confound the benefit of computer-assisted technologies in improving enophthalmos in this study.

Post-traumatic enophthalmos is not only related to orbital volume expansion but also loss of ligamental support, soft tissue atrophy and contracture^36^. However, current paradigm of post-traumatic orbital reconstruction focuses on restoring bony orbital volume and shape, which were found to correlate better with postoperative persistent enophthalmos than soft tissue atrophy in the long run^37^,^38^,^39^,^31^. Yet the latter may explain why anatomical reduction of the orbital fracture alone does not always guarantee long-term symmetry^40^. Soft tissue changes, if any, may be evaluated by systematically comparing magnetic resonance images (MRI) from both injured and uninjured orbits of surgically and conservatively managed patients^41^.

Post-traumatic diplopia can be caused by direct nerve or muscle injury causing paralytic strabismus or herniation/entrapment of muscle and/or surrounding connective tissue leading to restrictive strabismus. Our results showed that computer-assisted technology may provide additional improvements in diplopia compared to the conventional surgery. Fewer patients undergoing computer-assisted repair had residual diplopia except those in the study of Lauer *et al.*^21^. In that study, the authors used polydioxanone sheets (PDS) implants for 75% and 86% of cases in the assisted and control group respectively. Large orbital defects (>2.5 cm^2^) were present in 85% in the assisted group and 88% in the control group. However, previous study showed that PDS implant was suitable only for smaller defects (<2.5 cm^2^)^42^, thus potentially undermining the beneficial effect of intraoperative navigation^21^. We postulate that using computer-assisted technology allows more accurate anatomical restoration with better soft tissues repositioning and less implant impingement, leading to better postoperative motility and less diplopia.

Post-operative complications were similar in both groups with no statistical difference. These complications were inherent to the complexities and surgical approaches rather than the use of computer-assisted technologies.

Revisions surgeries are more challenging as scarring occurs over inadequately reduced soft tissues, remaining bone fragments as well as malpositioned implants. Extensive dissection and osteotomies are often necessary and spatial orientation and visualization become more difficult^43^. Secondary reconstruction is thus one of the strongest indications in using computer-assisted technologies. However, the two studies on secondary reconstruction did not show any additional benefit^21^,^22^.

### Recommendations for future studies

Existing evidence is deemed insufficient to determine the emerging role of computer-assisted technologies. Future randomized trials will have to address important confounders including size and pattern (e.g. isolated floor versus combined medial wall and floor) of fracture, timing of repair (immediate versus delayed), types of implant and number of surgeons involved. It would also be relevant to study its impact among surgeons-in-training and in revision or complicated cases. Extended follow-up will allow evaluation of soft tissue atrophy, fibrosis and contracture on late-onset diplopia and enophthalmos. Finally, direct and indirect costs should be included in future studies.

## Conclusion

To our knowledge, this is the first systematic review on computer-assisted technologies for post-traumatic orbital reconstruction. Results are limited by the paucity of high-quality studies available. Our data suggests advantage in its use for treating diplopia but not enophthalmos. As attractive and innovative as other new technologies, it is not a prerequisite for good outcomes, particularly in simple cases. Despite the potential to reduce error and subjectivity, it is still far from being routinely used due to the incurred time and cost. This review highlights the need of carefully-designed, randomized controlled trials in assessing the indications, safety and efficacy, as well as cost utility of computer-assisted technologies in post-traumatic orbital reconstruction.

## Additional Information

**How to cite this article**: Wan, K. H. *et al.* The Role of Computer-Assisted Technology in Post-Traumatic Orbital Reconstruction: A PRISMA-driven Systematic Review. *Sci. Rep.* **5**, 17914; doi: 10.1038/srep17914 (2015).

## Supplementary Material

Supplementary Information

## Figures and Tables

**Table 1:** Characteristics of Included Trials.

Study	Year	Study Design	Patients/Orbits (n)	Mean FU duration (range), months	Fracture Patterns	Primary or Secondary Reconstruction
Bly^19^	2013	HCT	90/90	257 days (8–1209 days)	*Assisted*: Floor (18%), Medial Wall (7%), 2 Walls (38%), 3 or 4 Walls (38%) *Control*: Floor (20%), Medial Wall (4%), 2 Walls (33%), 3 or 4 Walls (42%)	Primary
Cai^20^	2012	PCS	58/58	12	*Assisted*: Floor (44%), Roof (8%), Medial wall (24%), Lateral wall (24%) *Control*: Floor (49%), Roof (7%), Medial wall (22%), Lateral wall (22%)	Primary
Lauer^21^	2006	PCS	20/24	(12–36)	≥3 of the following involved: zygomatic, floor, medial wall, roof, naso-orbito- ethmoid complex, maxilla	Secondary
Nkenke^22^	2011	PCS	20/20	(3–12)	Floor	Secondary
Scolozzi^23^	2010	PCS	20/20	(6–13)	*Assisted*: Medial wall (0%), Floor (70%), Both (30%) *Control*: Medial wall (30%), Floor (60%), Both (10%)	Primary
Guo^24^	2009	RCS	61/61	14.6 (8–22)	Floor	Primary

FU = follow up; HCT = Historically Control Trial; PCS = Prospective Cohort Study; RCS = Retrospective Cohort Study.

**Table 2:** Details of the Operative Techniques, Surgical Approach and Implants Used.

Study	Study Objective (Assisted vs Conventional)	Technique	Surgical Approach	Implants
Bly^19^	preoperative reconstruction with MIO with endoscopic intraoperative navigation device vs unassisted	MIO + endoscopic intraoperative navigation (iNtellect Cranial Navigation, Stryker Corp, Michigan, USA)	TC, inferior fornix, precaruncular, or lateral retrocanthal, depending on location of fracture	non-preformed, mostly titanium mesh coated with high-density polyethylene, followed by bare titanium mesh, PDS sheet was rarely used
Cai^20^	preoperative reconstruction with MIO with intraoperative navigation device vs unassisted	MIO + intraoperative navigation (iPlan Cranial,version 2.6; Brainlab, Feldkirchen, Germany)	N/A (assisted and control were matched for surgical approach)	N/A (assisted and control were matched for implants used)
Lauer^21^	intraoperative navigation device vs unassisted	intraoperative navigation (Vector Visions^2^, BrainLab, Heimstetten, Germany)	Mostly coronal, infraorbital, and intraoral; medial TC or medial eyebrow incision for isolated medial wall, 3 used preexisting scars and intraoral	non-preformed, 60% PDS, 25% none, 10% calvarial bone plus PDS, 5% titanium mesh
Nkenke^22^	individualized preformed CAD/CAM glass-bioceramic implants vs non-preformed titanium mesh	No intraoperative navigation used	Assisted: 60% subciliary, 30% TC, 10% TC + lateral canthotomy; Control: 50% subciliary, 50% TC	preformed CAD/CAM glass-bioceramic, non-preformed titanium mesh
Scolozzi^23^	individualized preformed CAD/CAM titanium mesh vs non-preformed titanium mesh	No intraoperative navigation used	N/A	preformed titanium mesh, non-preformed titanium mesh
Guo^24^	individualized preformed CAD/CAM titanium mesh vs calvarial bone	No intraoperative navigation used	TC	preformed titanium mesh, non-preformed calvarial bone

CAD = computer- assisted designed; CAM = computer-assisted manufactured; MIO = mirror image overlay; TC = Transconjunctival; PDS = polydioxanone sheets; N/A = not available.

**Table 3:** Quality of Included Studies, Using the Newcastle-Ottawa Quality Assessment Scale for Cohort Studies.

Studies	Selection				Comparability	Outcome			Quality score (Number of stars)
	Representativeness of the exposed cohort	Selection of the non exposed cohort	Ascertainment of exposure	Demonstration that outcome of interest was not present at start of study	Comparability of cohorts on the basis of the design or analysis	Assessment of outcome	Was follow up long enough for outcomes to occur	Adequacy of follow up of cohorts	
Nkenke^22^	*	-	*	*	*	*	*	*	7
Guo^24^	*	-	*	*	-	*	*	*	6
Cai^20^	*	*	*	*	**	*	*	*	9
Scolozzi^23^	*	*	*	*	-	*	*	*	7
Lauer^21^	*	*	*	*	-	*	*	*	7

**Table 4:** Quality of Included Studies, Using the Newcastle-Ottawa Quality Assessment Scale for Case Control Studies.

Studies	Selection				Comparability	Exposure			Quality score (Number of stars)
	Is the case definition adequate	Representativeness of the cases	Selection of Controls	Definition of Controls	Comparability of cases and controls on the basis of the design or analysis	Ascertainment of exposure	Same method of ascertainment for cases and controls	Non - Response rate	
Bly^19^	*	*	*	*	*	*	*	*	8

**Table 5:** Summary of outcomes of the three studies comparing image guided surgery with control.

Study	% of patients experiencing residual diplopia	% of patients having residual enophthalmos	Complications
Bly^19^	Assisted: 51%Control: 60%(p = 0.003)	N/A	Assisted: 2 extrusion or infection requiring implant removal, 2 entropion, 2 ectropion, 1 epiphora Control: 3 extrusion or infection requiring implant removal, 1 eyelid abscess, 1 retrobulbar hematoma (p = N.S.)
Cai^20^	Assisted: 2%Control: 10%(p = 0.039)	Assisted: 3%Control: 10%(p = 0.625)*	N/A
Lauer^21^	Assisted: 86%Control: 25%(p = N.S.)	Assisted: 27%Control: 40%(p = N.S.)*	Assisted: 0Control: 0(p = N.S.)

N/A = not available; N.S. = non-significant; *exophthalmometry data not reported.

**Table 6:** Summary of outcomes of the three studies evaluating individualized preformed implants.

Study	% of patients experiencing residual diplopia	Enophthalmos	Complications
Nkenke^22^	N/A	Reduction in Enophthalmos (mm) ± SD Assisted: 3.25 ± 1.44 Control: 3.88 ± 1.23 (p = 0.31)	Assisted: 1 reduced vision, 2 suspected retrobulbar hematoma Control: 2 suspected retrobulbar hematoma (p = N.S.)
Scolozzi^23^	N/A	Reduction in Enophthalmos (mm) ± SD Assisted: 1.5 ± 1.58 Control: 1.8 ± 1.61 (p = 0.79)	N/A
Guo^24^	Assisted: 17% Control: 88% (p = 0.0042)	Assisted: 21%* Control: 50%* (p = 0.04)*	N/A

N/A = not available; N.S. = non-significant; *percentage of patients having residual enophthalmos; *exophthalmometry data not reported; SD = standard deviation.
